# Editorial: Molecular ecology of plant sexual reproduction

**DOI:** 10.3389/fpls.2024.1362777

**Published:** 2024-01-24

**Authors:** Yuan-Wen Duan, Ming-Xun Ren, Yan-Bing Gong, Bin Tian, Juli Caujapé-Castells

**Affiliations:** ^1^ Germplasm Bank of Wild Species, Institute of Tibetan Plateau Research at Kunming, Kunming Institute of Botany, Chinese Academy of Sciences, Kunming, Yunnan, China; ^2^ Key Laboratory of Genetics and Germplasm Innovation of Tropical Special Forest Trees and Ornamental Plants, Ministry of Education, Hainan University, Haikou, China; ^3^ Key Laboratory of Biodiversity and Environment on the Qinghai-Tibetan Plateau, Ministry of Education, College of Life Sciences, Wuhan University, Wuhan, China; ^4^ National Plateau Wetlands Research Center, Southwest Forestry University, Kunming, China; ^5^ Jardín Botánico Canario ‘Viera y Clavijo’-Unidad Asociada CSIC, Cabildo de Gran Canaria, Las Palmas de Gran Canaria, Spain

**Keywords:** heterospecific pollen transfer, style dimorphism, pollination network, sex determination, genomic era

Sexual reproduction plays a crucial role in the life history of plants, and the relationship between plants and their pollinators is a significant aspect of plant sexual reproduction because more than 80% of angiosperms rely on animal pollinators for seed production (Ollerton et al., 2011; Tong et al., 2023). Currently, the rapid advancements in DNA sequencing over the past decade have fueled researches on the evolutionary outcomes of shifts in mating systems (Wang et al., 2021; Wu et al., 2022), and the molecular mechanisms underlying floral evolution (Todesco et al., 2022; Gao and Chen, 2023; Zhou et al., 2023). It is evident that next-generation sequencing will certainly contribute to advancing our understanding of plant sexual reproduction. This Research Topic is composed of 4 articles on molecular ecology of plant sexual reproduction, with aims to applying molecular tools in studying plant sexual reproduction.

## Convergent sex determination

Dioecy, a sexual system consisting of separate male and female plants within a single population, is present in 5-6% of flowering plants across various plant lineages (Renner, 2014). It offers an opportunity to investigate potential convergent mechanisms. The rapid advancement of genome sequencing has led to the discovery of convergent evolution in sex determination among dioecious plants (Liu et al., 2022). Zhang et al. conducted a comprehensive review of the progress made in understanding sex chromosomes, sex-determining genes, and floral MADS-box genes in dioecious plants. In dioecious plant species that have been sequenced, sex chromosomes were identified in only 33 species belonging to 22 families. While the specific sex-determining genes may vary among different species, they converge on floral MADS-box genes and B-class genes, resulting in a convergent model for sex determination. This review provides valuable insights for a comprehensive understanding of the mechanisms underlying sex determination and promotes the application of sex regulatory genes in breeding programs for economically important dioecious plant species.

## Effects of heterospecific pollen transfer

In a plant community, when animal pollinators visit co-flowering plants, they can facilitate both interspecific and conspecific pollen transfer (Moreira-Hernandez and Muchhala, 2019). Interspecific pollen transfer, also known as heterospecific pollen transfer, can lead to reduced reproductive success via stigma clogging, stigma closure, pollen competition and allelopathy in plant species with incompatible pollen grains (Morales and Traveset, 2008). For plant species with compatible pollen grains, reproductive fitness could be decreased due to stylar clogging and ovule wastage resulting from hybridization (Liao et al., 2019). However, it remains unclear how co-flowering congenic plant species maintain their reproductive fitness in the presence of heterospecific pollen transfer. Fei et al. investigated the advantage of conspecific pollen and its ability to resist interspecific reproductive interference between two *Sagittaria* species that are capable of producing hybridized seeds through reciprocal pollination. By using the nrITS sequence for the identification of hybridized seeds, they concluded that in both species, congenic pollen outcompeted heterospecific pollen, facilitated by the extragynoecial compitum. This study enhances our understanding of how closely related plant species maintain their species integrity within a community.

## Mechanism underlying style dimorphism

Heterostyly, characterized by two (distyly) or three (tristyly) floral morphs that differ reciprocally in the relative positions of stigmas and anthers within a species, is maintained through a strong diallelic self-incompatibility system. In recent years, candidate genes underlying heterostyly have been identified associated, and the inactivation of brassinosteroids (BR) may be a key genetic mechanism underlying style-length regulation in species such as in *Primula veris* (Primulaceae), *Gelsemium elegans* (Gelsemiaceae) and *Turnera* (Passifloraceae) (Zhao et al., 2023). However, the genetic basis of style dimorphism, a trait characterized by different style heights but similar anther heights, often termed “anomalous” distyly or stigma-height dimorphism (Barrett et al., 2000), remains poorly understood. Luo et al. compared transcriptomic profiling of styles and androecium in different developmental stages between long- and short-styled flowers of *Guettarda speciosa*, a tropical tree species exhibiting style dimorphism. The findings revealed that BKI1, a negative regulator of brassinosteroid signaling, was up-regulated in the styles of the S-morph, potentially indicating its involvement in the regulation of style length in *G. speciosa* through a BR-related signaling network. These results demonstrated that style length in *G. speciosa* with “anomalous” distyly is influenced by differential gene expression, in contrast to the S-locus genes observed in typical distylous flowers.

## Floral traits and pollination network

On the community level, pollination networks are characterized by modularity, nestedness, asymmetry, and spatial and temporal variation (Fang and Huang, 2012). Floral traits play a crucial role in influencing pollinator diversity, which in turn affects the community-level pollinator network (Suarez-Marino et al., 2022). Xiang et al. investigated the phylogenetic signals of floral traits and their impact on pollination networks in a community, with an aim to examine how floral traits influence the structure of pollination networks. They discovered significant phylogenetic signals in all floral traits, except for flower density. Plant specialization and its contribution to network modularity were found to be influenced by both floral size and floral density, although the effect of flower size was more complex than floral density. Plant species with larger flowers tended to occupy central roles in the nested and modular pollination networks. Additionally, floral shape, symmetry, and color were identified as factors that contribute to the co-flowering filters in pollination sharing and help shape network modularity. These findings highlight the importance of phylogenetically conserved traits, known as pollination syndromes, as drivers for the modular structure of local pollination networks.

In this Research Topic, the number of received articles was far less than we expected. Clearly, application of molecular methods as tools in pollination biology could help us in deciphering mechanisms underlying mating/sexual system shift, flower evolution and plant-pollinator relationships, which would undoubtedly advance our understandings on plant sexual reproduction in genomic Era.

## Author contributions

Y-WD: Funding acquisition, Validation, Writing – original draft. M-XR: Conceptualization, Funding acquisition, Validation, Writing – review & editing. Y-BG: Conceptualization, Validation, Writing – review & editing. BT: Conceptualization, Validation, Writing – review & editing. J-CC: Validation, Writing – review & editing.
